# Aptitude of Oxidative Enzymes for Treatment of Wastewater Pollutants: A Laccase Perspective

**DOI:** 10.3390/molecules24112064

**Published:** 2019-05-30

**Authors:** John O. Unuofin, Anthony I. Okoh, Uchechukwu U. Nwodo

**Affiliations:** 1 SAMRC Microbial Water Quality Monitoring Centre, University of Fort Hare, Private Bag X1314, Alice 5700, South Africa; AOkoh@ufh.ac.za (A.I.O.); UNwodo@ufh.ac.za (U.U.N.); 2 Applied and Environmental Microbiology Research Group (AEMREG), Department of Biochemistry and Microbiology, University of Fort Hare, Private Bag X1314, Alice 5700, South Africa

**Keywords:** wastewater pollutants, wastewater treatment, oxidative enzymes, laccase, environomics

## Abstract

Natural water sources are very often contaminated by municipal wastewater discharges which contain either of xenobiotic pollutants and their sometimes more toxic degradation products, or both, which frustrates the universal millenium development goal of provision of the relatively scarce pristine freshwater to water-scarce and -stressed communities, in order to augment their socioeconomic well-being. Seeing that both regulatory measures, as regards the discharge limits of wastewater, and the query for efficient treatment methods remain unanswered, partially, the prospects of enzymatic treatment of wastewater is advisable. Therefore, a reconsideration was assigned to the possible capacity of oxidative enzymes and the respective challenges encountered during their applications in wastewater treatment, and ultimately, the prospects of laccase, a polyphenol oxidase that oxidizes aromatic and inorganic substrates with electron-donating groups in treatment aromatic contaminants of wastewater, in real wastewater situations, since it is assumed to be a vehicle for a greener community. Furthermore, the importance of laccase-driven catalysis toward maintaining mass-energy balance, hence minimizing environmental waste, was comprehensibly elucidated, as well the strategic positioning of laccase in a model wastewater treatment facility for effective treatment of wastewater contaminants.

## 1. Introduction

The universal epiphany that wastewater might still serve as thresholds for heterogeneous blends of surreptitious but sinister chemicals in our natural water bodies has led to conscientious efforts toward mitigation by several communities, some of which are: prevention of untreated waste disposal, and possibly, the control of their sources, separation of wastewater with regard to their sources and regionalized treatments plants [1,2]; the latter two are run by treatment technologies based on physico-chemical approaches in wastewater treatment plants (WWTPs) whose operation require technical skills, high operation costs (in terms of high energy and chemical demand). Wastewater treatment is traditionally employed to protect the quality of limited freshwater resources, which are most times the final discharge points of effluents, and also, to promote the reusability of expended clean water; notwithstanding, noticeable amounts of hazardous aromatic byproducts are still generated [3,4]. This stems from the observation that conventional wastewater treatment plants, though liable to sparingly remove microcontaminants such as heavy metals, and to a far lesser extent, aromatic contaminants, were originally structured for the removal of solid wastes, ecofriendly organic matter and eutrophication stimulants from wastewater, thereby reducing eutrophicating pollution loads; albeit the micropollutants may only be moderately affected by the chemical, physical and biological interactions within the treatment plants. These characteristics therefore make wastewater treatment a more complicated phenomenon than water treatment itself, hence wastewater must be thoroughly treated before it is regarded as innocuous for discharge back into the environment [5].

So far, a number of physico-chemical techniques have been exploited, *viz* coagulation, flocculation and activated carbon adsorption; more sophisticated technologies, such as reverse osmosis, nanofiltration, photolysis, ion exchange and advanced oxidation processes posit successes at the laboratory scale but are still too expensive for demonstration of real life treatment of municipal wastewaters. Moreover, advanced technologies often require the expertise of trained technicians, who in most cases are permanent on-site staff, and they consume signifcant energy, and potentially, non-renewables, which sabotages the efforts of green technology.

The use of oxidation reactions, though effective and essential in treatment of wastewater, could be impeded by drawbacks, such as non-specificity, undesirable by-products and use of environmentally malignant chemicals, which are encountered when using conventional oxidation technologies. An example is the conferral of objectionable odour and taste to phenol-containing water when disinfected with chlorine, a potent oxidizing agent [6,7] due to the formation of halophenols and haloaromatics as byproducts [8]. Biological processes such as the activated sludge and trickling filters have been considered as more beneficial than the previously mentioned methods, due to their effectivity and the formation of relatively non-toxic end products. However, researchers in time past have warned against the absolute dependence on these biological methods, because of various physiological, biochemical and ecological challenges associated with their application [9,10]. Furthermore, they are unable to eliminate a broad range of emerging contaminants, most of which remain soluble in the effluent [11,12,13]. Altogether, the occurence of emerging contaminants in wastewater has rendered the relative efficiencies of these treatment processes mediocre, owing to the surreptitiousness of majority of these compounds, and as such, regulatory bodies have had difficulties in establishing discharge limits and concentrations to the spiraling priority list of these contaminants.

This has motivated the heuristic search for innovative oxidation technologies, which are enzyme-driven [14]. The use of enzymes presents a tremendous alternative, since they lower the activation energy by bringing reactants closer—a process that may weaken chemical bonds— thereby making reactions proceed faster, and their indestructibility in the reactions which they catalyze. Among the enzymes secreted in Nature, the oxidoreductases have been regarded as highly relevant in many industries due to their ability to transfer electrons from one substrate molecule to another. Members of this class include dehydrogenases, reductases and the oxidases. Well over 200 kinds of oxidases have been discovered so far, six amongst which are capable of using the unique redox ability of copper ions to catalyze the oxidation of varied aromatic substances. Enzymes belonging to these members, being electron shuttles, participate either in the modification and/or the complete substitution of conventional methods of operation in the industries. In particular, foremost attention is given to an interesting cluster of oxidative enzymes, whose utilization as biocatalysts was previously neglected due to their unavailability on commercial scale, “laccases” [15].

This review therefore gives a terse account on wastewater treatment modes applied so far and the effect of the greener and more economical mode of treatment, “bacterial laccase” on these contaminants in wastewater treatment.

## 2. Existing Treatment Technologies for Wastewater: Towards Greener Oxidations

In a bid to safeguard the aquatic ecosystem and to sustain drinking water resources, alleviation of the presence of of organic pollutants in surface waters in imperative. Although prevention and source control, source separation and decentralized treatment or centralized end-of pipe treatments are essential levels of actions enforced in obviating the occurence of organic pollutants in our freshwater resources [1,2], more action should be channelled towards the mode of operation in the wastewater treatment facility. Altogether, to tackle water-quality issues arising from wastewater organic pollutants, cost-friendly treatment technologies must be promoted. Several methods for wastewater treatment have been proposed, which relate to physical, chemical and biological methods. Physical methods target solid or liquid pollutant removal based on their density gradients. In a wastewater treatment plant, they are employed to get rid of colloids, sludge or liquids. Such effluent treatment methods include reverse osmosis, electrodialysis, filtration, foam separation, porous bed filtration, adsorption *inter alia*. They aid in the removal of fine particles, dissolved materials (both organic and inorganic), thereby ensuing better water quality for subsequent re-use or discharge. The chemical methods include treatments like oxidative processes, ozonation, photochemical and electrochemical destruction. Given the drawbracks; high cost, high sludge production, formation of toxic byproducts, etc. suffered by the prevalent physico-chemical methods of effluent treatment, and the encumbrance experienced while employing the more efficient advanced physico-chemical treatments, such as expensiveness, especially for small installations, requirement of adequate technical expertise of a permanent staff for effective operation [2], consumption of significant energy, and potentially, non-renewable fossil fuels, which in the long run invalidate any efforts made towards the reversal of climate change, and so forth. Moreover, upsetting concentrations of organic pollutants have been found in water caches receiving large amounts of wastewater treatment plant effluents; a consequence of the low dilution of the effluents [16].

Biological methods of wastewater treatment have received much attention in recent years. Their increasing importance is by virtue of the possibility of total oxidation and sanitation of many impurities, including toxic ones, while requiring comparatively low operating cost and simple equipments, through microorganisms or their metabolites. Where microorganisms are adopted, recalcitrant, xenobiotic and highly resistant natural molecules such as aromatic contaminants could be utilized as nutritive substrates, where benign oxidation products such as H_2_O and CO_2_ are anticipated as a result of their assimilation [17,18,19]. However, this method is relatively slow and its efficiency would hinge on the maintenance of the microbial consortia employed in biodegradation. Correspondingly, our deductions from a compilation of different extant wastewater treatment techniques reflected in a recent exhaustive review by Crini and Lichtfouse [20] suggest their futility, especially when their gross comparative effects are examined. These disadvantages therefore necessitate the need for developing alternate effluent treatment methods with marginal needs of equipment, maintenance, technique and energy; hence the significance of biological methods of effluent treatment. Conversely, the employment of microbial metabolites involves, predominantly, oxidative enzymes that catalyze oxidation-reduction reactions: the oxidoreductases. These biomolecules exhibit broad substrate specificity, regio- and enantioselectivity, and are effective in the treatment of heterogeneous effluents, especially dyes, phenols and related compounds that are normally recalcitrant to bacterial degradation at high concentrations [21,22].

Oxidative enzymes, unlike many chemical oxidants, do not necessarily mineralize the substrate, instead they form radicals, which could be disintegrated to fractions of transformation products, or be coupled with different molecules, through non-enzymatic processes (oxidative coupling reactions), to form higher molecular weight compounds [23]. More frequently, the transformation products of enzyme-catalyzed treatments were reported to exhibit minimal toxicity or were more biodegradable than the parent compounds, proving the aptitude of biological oxidative treatment to embelish wastewater condition [24,25,26,27,28,29,30,31]. The intervention of innovative biological oxidation processes (using oxidative enzymes), with low energy- and resource throughput, although demanding probably longer reaction times and larger space, may be an attractive route for micropollutants treatment in miniature wastewater treatment plants, where size is negligible.

## 3. Oxidative Enzymes

The past decade had recorded explicit reviews on the potentials of oxygen scavenging enzymes from bacterial, plant and fungal origin in the removal of environmental pollutants and microcontaminants. Some famously reported families are the oxygenases, peroxidases and polyphenol oxidases [14,32,33,34,35] which all initiate the disintegration of phenolic, aromatic or inorganic pollutants *via* oxidation mechanisms. The properties of these enzyme batteries, which make them aid in the metabolism of otherwise recalcitrant sources of carbon or energy [36] by various groups of microorganims, are briefly highlighted *infra*.

### 3.1. Oxygenases

Oxygenases are a class of mostly intracellular enzymes that perform functions crucial to biosynthesis and metabolism. Apart from their use in the fabrication of pharmaceuticals and the synthesis of specialty chemicals [37], they derive relevance in the biodegradation of hydrocarbons and their analogous compounds [38] that have been considered environmental pollutants, consequent of their improvident use, storage and disposal: a process generally regarded as biotransformation. Correspondingly, biotransformation *via* oxygenases is consdered ideal for three salient reasons among other things:

- The non-existence of their chemical counterparts or lack of the requisite regio- and stereoselectivity.
- The availability of molecular oxygen as a cheap environmentally benign oxidant in contrast to harsh chemical oxidants [39].
- Their participation in the modification of natural products or synthesis of pharmaceutical intermediate or chiral building blocks [37].

Oxygenases accomplish the regio-, stereo- and enantioselective introduction atomic or molecular oxygen into an astounding range of susbtrates, thereby converting hydrophobic compounds, which are mostly of endobiotic and xenobiotic origin, into more water-soluble and responsive forms [40,41]. Mason and Commack [42] suggested that the various oxygenases could be distinguished based on their structure, mechanism and cofactor requirement. However in 2014, Jadeja and co workers [43], after a conclusive study claimed that the innate function of oxygenases is well aligned, irrespective of their geographical location or environmental niches. In the posterior half of the previous century, their importance in the activated sludge step of secondary wastewater treatment was highlighted, where they were perceived to add hydroxyl groups to the carbon backbone or carboniferous organic, aromatic pollutants, thereby stimulating their oxidation [44]. Since the triplet state (^3^O_2_) in which molecular oxygen exists makes it kinetically stable and unreactive, due to the presence of two unpaired electrons [45], this could slow down the oxidation of organic compounds as to be almost biologically insignificant, notwithstanding their thermodynamic tangibility, if carried out in the absence of a catalyst. Oxygenases therefore surmount this circumstance: by either making oxygen more reactive through O_2_ activation, or making the substrate more susceptible to attack by O_2_ (substrate activation). The accomplishment of these phenomena is attributed to the scantily occupied electron or radicals or transition metal ions in the mould of mononuclear, binuclaer or haem center, or an organic cofactor (electron transport entities), which accelerates the reaction. Iron and copper have been recognized as the most exploitable metals in this context because their lower oxidation states form complexes with O_2_ and/or substrate, resulting in electronic structure modification of the bound moiety, and ultimately altering its reactivity. Therefore, based on oxygen utilization, oxygenases can be grouped as monooxygenases and dioxygenases. The monooxygenases direct the solitary reduction of atomic oxygen that subsequently appears as the addition of an individual hydroxyl group, which is often cofactor-dependent, whereas the dioxygenases catalyze the synchronous stereospecific reduction of two oxygen atoms, which translates into the addition of *cis* oriented hydroxyls to an aromatic ring [46]. However, the development of oxygenases for industrial purposes faces hurdles that are unusual amongst other biocatalytic processes: they are often unstable, consist of several constituents, some of which might be membrane-bound hence requiring expensive cofactors such as NAD(P)H [47,48]. These limitations might only be overcome by using whole-cell systems. Owing to the relatively high *K_m_* of some oxygenases for oxygen, it might be necessary to maintain significant oxygen pressure during bioconversion to allow the oxygenase to compete for oxygen with the electron transport chain [47]. Furthermore, although small hydrophobic compounds are readily absorbed by the cell biocatalysts, as potential substrates for oxygenases, larger or charged molecules might not easily get into the cell, thereby making the detection of enzyme-producing strains somewhat queasy due to the unavailability of these substrates for oxidation during screening, hence yielding false negatives [40].

### 3.2. Peroxidases

Peroxidases, sometimes reffered to as heme-containing proteins (although not exclusively) are vastly present in all forms of life; from the decomposers and producers, to the consumers. They have been reported to prevent oxidative damage to plant leaves [49] and contribute to the lignification process [50]. They mediate the reduction of peroxides, notably hydrogen peroxide (H_2_O_2_) or any organic peroxide, with concomitant oxidation of chemically diverse compounds. In spite of their unanimous requirement for hydrogen peroxide as co-substrates, they could be distinguished based on their in-depth mode of action, and possibly, the number of isoforms secreted during nutrient limiting conditions [51]. A classical example refers to reports of Burton [33] and Oyadomari et al. [52], who asserted lignin peroxidase to be the only known extracellular peroxidase capable of oxidizing non-phenolic aromatic substrates with high redox potential, while Karam and Nicell [53] identified the stringent requirement of high manganese ions and chelators, asides hydrogen peroxide, as the limitation of wastewater treatment using manganese peroxidase. Peroxidase activity precisely entails the transfer of electrons to substrates such as ferricyanides, so that they are disintegrated into innocuous constituents in reactions, which are are characteristic of any of the following four categories as described by van Duerzen et al [54]:

- Oxidative dehydrogenation: this reaction is delimited to heme peroxidases, and it employs a one-electron transfer, where radical cations and radicals participate as intermediates, resulting in inter- or intra-molecular radical coupling derivatives. An example is the dealkylation of heteroatoms.
- Oxidative halogenation: although not circumscribed to heme peroxidases, it is understood to be initiated by active halide species. Its peroxidase-driven reactions are, by and large, regarded non-selective and occur outside the active site. Wiesner et al. [55] however presented an exception, where indole was regioselectively converted to 7-chloroindole by a metal-free bacterial chloroperoxidase.
- H_2_O_2_ disproportionation: here, H_2_O_2_ is disintegrated into water and oxygen, either directly or by the reaction of hydrogen peroxide with hypohalous acid to generate atomic oxygen.
- Oxygen transfer reactions: it entails the selective transfer of oxygen, which is somewhat similar to the action orchestrated by mono-oxygenases. Such reactions are represented by the following: Heteroatom (sulfur and nitrogen) oxidation, epoxidation and CH bond oxidation.

The aforementioned reactions have accorded peroxidases centrality in the treatment of recalcitrant heterogenous wastewater contaminants; dyes, phenols and aromatics, which are sourced from domestic and industrial effluents. Contrariwise, the rapid voluntary inactivation by excess H_2_O_2_ and the speedy expenditure of H_2_O_2_ by the wastewater milieu, due to their interaction with organic matter [56,57] make them unsuitable for real wastewater treatments. This could be due to the possibility of a sharp decline in enzyme activity or the incessant demand for high H_2_O_2_ concentrations. So far, only four peroxidases have been comprehensively studied; details on peroxidases could be retrieved from reviews by Welinder [51], van Duerzen et al. [54], van Rantwijk and Sheldon [58], Husain [59] and Bansal and Kawar [60].

### 3.3. Polyphenol Oxidases

Polyphenol oxidases are a group of binucleate cupriferrous enzymes that have been thought to participate in the suitability and objectionableness of certain commercial edibles; from the desirable browning of tea, coffee and cocoa to the undesirable browning effects on certain fruits, vegetables and processed wines and beverages. They are extensively exploited in several applications, which are not limited to enhancement of flavour [61,62], determination of food quality [63,64], and the removal phenolic pollutants from wastewater [65]. They achieve these by the hydroxylation of aromatic rings, followed by a subsequent oxidation of the mono- or diphenols generated (usuallly an oxygen-reliant reaction) to highly reactive quinones and radicals, which may further undergo a non-enzymatic polymerization, or form high insoluble molecular pigments on reaction with other substances [66,67,68]. They are almost ubiquitous in nature and their secretion is mostly associated with pathogenic functions in fungi, and physiological responses to pertubations such as stress and mechanical injury in plant tissues. On account of extensiveness of study and the types of phenolic compounds which they oxidize, they are delineated into two main groups: tyrosinases and laccases.

#### 3.3.1. Tyrosinases

Tyrosinases are type-3 cuprodinucleate metalloproteins that catalyze oxidation reactions similar to the peroxidases, and are distinctively regarded for the crucial role they play in the synthesis of melanin pigment from l-tyrosine in animals as well as humans. Their cupriferous binuclear complex have been confirmed by spectral, crystallographic and chemical studies [69,70], which reveal that all tyrosinases, despite differences in structural conformation and molecular weight, posses a type-3 diamagnetically binucleated copper core of two copper atoms, each harmonized by three highly conserved histidine residues [71,72,73]. However, those of microbial sources are categorized based on domain organization and caddie protein requirement into five types as explained by Fairhead and Thöny-Meyer [74] and Zaidi et al. [73]. They are broadly circulated between and within species of a phylogenetic scale, where they serve bifunctional purposes [75,76]. They have been implicated in physiological, chemotrophic, pathogenic and photo- and radio-protective activities in plants, fungi and bacteria [77,78,79,80] with a characteristic three-dimensional structure first reported from *Streptomyces castaneoglobisporus* [70]. Conventionally, they oxidize their substrates by withdrawal of a pair of electrons, and their actvities are highlighted by two oxygen-contingent reactions: ortho oriented hydroxylation of monophenolic compounds to *o*-diphenols, which is characteristic of cresolase activity and the succeeding spontaneous oxidation of *o*-diphenols to *ortho*-adapted quinones, which is attributed to catecholase activity [72]. The reactive quinones undergo a non-enzymatic auto-polymerization [81,82] or are copolymerized with unsubstituted phenols to form insoluble agglomerates, which are less malevolent than the parent phenols [34], and are highly separable [83]. These mechanistic portraits should, quite expectedly, hypothetically elect tyrosinases as choice biomolecules for treatment of phenol-laden wastewater or soils. Surprisingly however, their comparatively lower redox potential [18], effortless inactivation in fluid matrix [84] and the miscibility of oxidized phenolic products [85,86] make them less fascinating for real largescale applications in the environment and industry. The reader is however directed to consult Claus and Decker [72], Chang [87], Fairhead and Thörny-Meyer [74], Kim et al. [88] and Zaidi et al. [73] and the patent review [89], including references therein for a comprehensive insight of selected subjects on tyrosinases.

The quest for more environmentally- and industrially-apposite enzymes, particularly those central to phenolic and aromatic pollutant alleviation in wastewater, has led to the reevaluation of laccases which, when compared to other enzymes adopted for phenolic and aromatic catalysis, are effortlessly accessible with improved stability, substrate speficity, not requiring exogenous supply of cofactors such as hydrogen peroxide (but may be enhaced by mediators) [33]. Thus, despite having redox potentials only preceded by the peroxidases, laccases are being proposed as interesting biocatalysts in various industrial and environmental modifications, principally wastewater management [17].

#### 3.3.2. Bacterial Laccases: A Choice Candidate

Ever since its initial discovery from the resin of the Japanese lacquer tree *Rhus vernicifera* (now *Toxicodendron vernicifluum*) in 1883 [90] in ancient China, laccase (EC 1.10.3.2) has consistently been detected in various plants [91,92,93] fungi [94,95,96], insects [97,98,99] and bacteria [100,101,102], where they play significant biological roles dictated oftentimes by physiological, anatomical, biochemical and chemotrophic orientation of their relative biota. In bacteria for example, it could be involved in morphogenesis, bioassimilation of recalcitrant aromatic substrates, pigmentation, sporulation, and could also form a protective shield against oxidizing agents and ultraviolet radiation [103] These proteins are copper-containing polyphenol oxidases, which catalyze the single electron-transfer oxidation of two pairs of reducing-substrate molecules, affiliated with the quadruplet electron reduction of molecular oxygen to water. Moreover, laccases also catalyze the oxidative cleavage of a broad range of substrates, especially phenolic compounds, but have some affinity for metal ions, some inorganic and organometallic complexes, and they differ from LiP and MnP on account of their unrequired exogenous supply of H_2_O_2_ or Mn^2+^, respectively, or other cofactors, for substrates cleavage. On the basis of substrate specificity among the polyphenol oxidases, only the laccases oxidize syringaldazine [104,105] making it an unparalleled indicator of laccase positive organisms in plate screens. Laccases have a substrate predilection in the order *ortho* > *para* > *meta* –substituted phenols [18], which could be broadened to include various aromatic compounds, especially those with electron-remitting groups like phenols (-OH) and anilines (-NH_2_), with the synchronous reduction of molecular oxygen to yield water [106]. The propensity of some laccases to oxidize diverse aromatics, a majority of which are of utmost health significance, has been reported in recent years [107,108,109,110,111,112]. The aforementioned qualities of laccases therefore imply their capability to initiate and, possibly, maintain the biodegradation of aromatic pollutants of wastewater, when applied in a supplementary treatment phase. Although comprehensively studied and produced using fungi as models, the extensive relevance of laccase in biotechnology has encouraged the expansion of spectrum of laccase producing microorganisms with essential physicochemical and catalytic properties [113]. While the wood degrading fungi are gaining much attention due to their production of copious amounts of extracellular lignolytic enzymes [114], marginal information depicts the aptitude of bacterial laccases in bioremediation trends. Moreover, bacteria have been considered more stable; given the tolerance of a broader range of habitats elicited by them, their faster proliferation rates [115], amenability to improvements in activity, selectivity, expression levels through protein engineering [116], and the likelihood of some of their laccases to be more thermostable and halotolerant [117,118,119]. These findings have been corroborated by the possibility that bacterial laccases are influential in the biodegradation of intractable biopolymers, as suggested by Ahmad et al. [120] and Bugg et al. [118] respectively.

**Figure 1:**
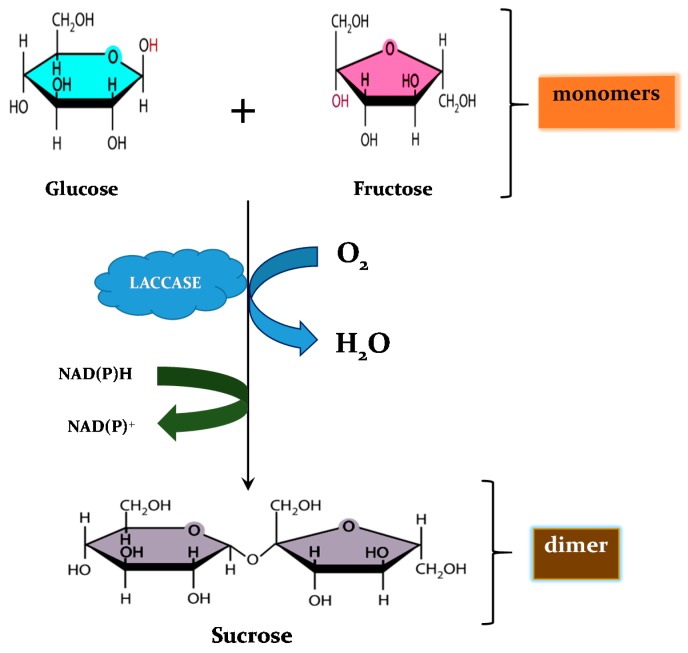
The hypothetical cross-linking of the fruit monomeric sugars, glucose and fructose to form the dimer sucrose or invert sugar.

**Figure 2:**
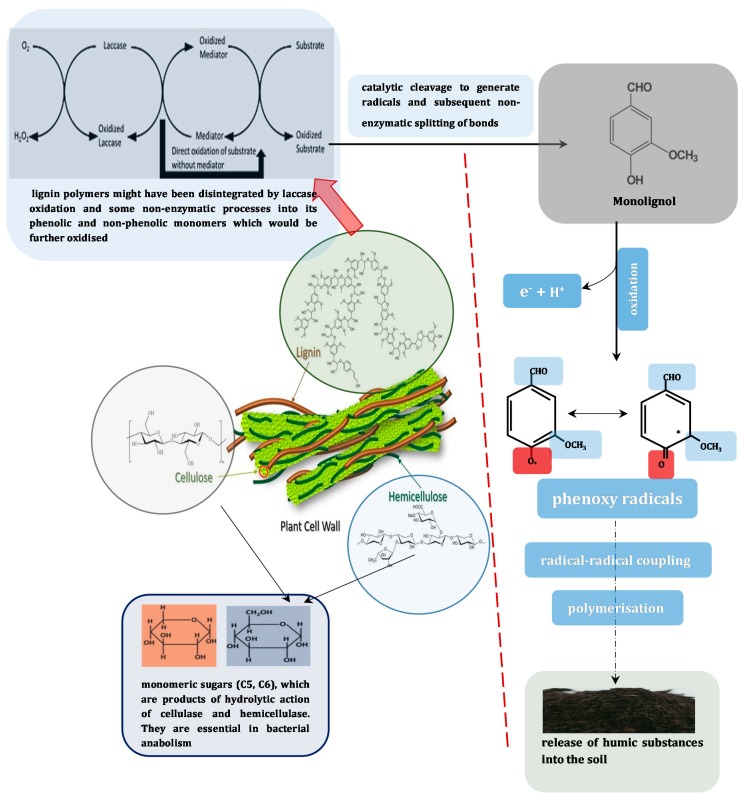
The characteristic disintegration of lignocellulosic polymers to liberate simpler carbon sources for bacterial metabolism and the consequent formation of humus from recalcitrant plant lignin residues and furfural. The left portion of the partition highlights the importance of hydrolytic enzymes in ensuring nutrient availability to the denizen bacteria, while the right portion summarises the mechanistic insight of humus formation.

**Figure 3:**
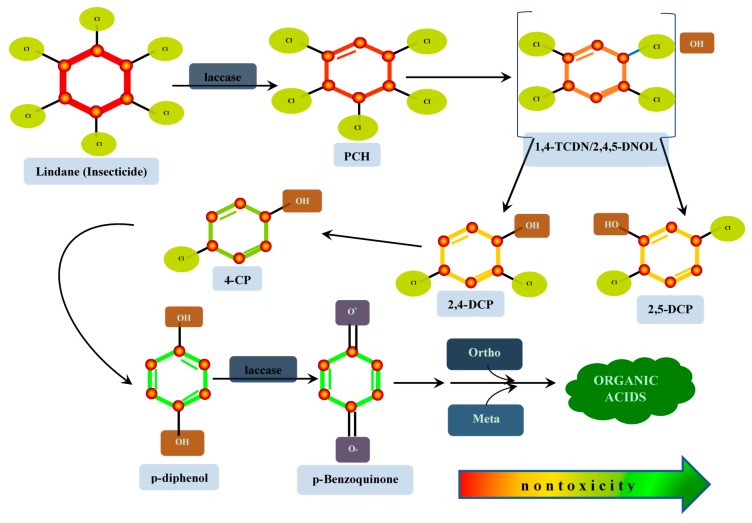
Hypothetical degradation of the insecticide, lindane, and its pathway to the production of organic acids. The colour tint on the rings represents the gradual metamorophosis from recaltrance and toxicity to biodegradability and eco-friendliness.

Whilst performing their catalytic routines, laccases redox transformations are summarized under the following:

(a) *Coupling of monomers*: where there is covalent linkage of C-C, C-O or C-N bonds of radicals generated by the oxidation of phenols and anilines, thereby forming dimers, oligomers or polymers. This process is exemplified by lignin polymerization and humic substance formation [109], or polymerization of melanin and spore coat resistance [121], and the novel hypothetical cross-linking of monomeric simple sugars in musts (Figure 1). It is noteworthy that this phenomenon can be employed in soil detoxification during which xenobiotics on soil agglomerates could be bound to the organic humic matrix.

(b) *Disintegration of polymers*: this could occur either during nutrient limiting conditions, where complex sources of energy and nutrients are employed as secondary substrates *via* mineralization, with the aid of oxidative enzymes, or during cometabolism, when they are fortuitously metabolized in the presence of simpler carbon and energy sources [122]. In the case of the first scenario, attack by laccases produces reactive radicals from these complex substrates, which drive the reduction of covalent forces in the attacked site, thereby releasing monomers. Many fungi, and some bacteria, participate actively in the humification of lignocellulosic biomass, especially when scarvenging for fibrous saccharide fragments embeded therein as source of carbon and other nutrients (Figure 2). This process has however been reported to be central to maintaining soil fertility [45].

(c) *Ring cleavage of alicyclic and aromatic compounds*: in this situation, atmospheric molecular oxygen is utilized by laccases in the primary transformation of xenobiotic compounds. Ring cleavage of alicyclics or aromatics is intiated by the transfer of molecular oxygen to cleave non-hydrolyzable C-C bonds of the rings, which drives its oxidative destruction to form unsaturated aliphatics, and ultimately, could generate intermediates of the cell metabolic cycle, depending on the parent structure of the xenobiotic (Figure 3). In our example, laccase catalyzes the dehydrochlorination of lindane to form pentachlorocyclohexene (PCCH). Pentachlorocyclohexene is converted to the unstable metabolite, 1,3,4,6-tetrachloro-1,4-cyclohexadiene (1,4-TCDN), which may be inter- convertible to 2,4,5-trichlorocyclohexenol (2,4,5-DNOL), and then spontaneously dechlorinates to form the isomers, 2,4-dichlorophenol (2,4-DCP) and 2,5-dichlorophenol (2,5-DCP). 2,4-DCP is thereafter dechlorinated by non-enzymatic electron transport processes to 4-chlorophenol (4-CP), which further undergoes an hydroxydechlorination to form *p*-diphenol. Here, laccase once again oxidizes the phenol to its corresponding quinone, which would follow any of the pathways (*ortho*/*meta*) for the synthesis of organic acids, which is a greener residue.

It is worth mentioning that the summarized activities supported by laccases, *vide* *supra*, are not exclusively laccase-driven, but could be aided by their oxidative *confrère*, reactions of radicals that are formed, and a number of intrinsic and environmental factors, which will be further expounded in following subsections.

##### Properties of Bacterial Laccases

Continuous debates and efforts have been made to understand the structural and functional properties of laccases; the first recorded attempt was made by Gabriel Bertrand [123], who, after relatively unsophisticated but adequate analytical methods, submitted that the metal, manganese, was an integral part as a ‘co-ferment’, better understood nowadays as a co-enzyme. This was critiqued in the twentieth century by some investigators who, although accepted his view of laccase being a metalloprotein, asserted that copper, and not manganese, was predominantly linked with the enzyme. Furthermore, his perceived oversight was attributed to the insufficient purity of his analyte [124]. Although coincidentally, another Bertrand [125], in conjunction with his co-investigators [126] stood by his namesake’s finds, due to a similar claim that his enzyme had a marginally higher presence of manganese relative to copper, a study by Tissieres four years later, with assistance from Keilin, reaffirmed laccase as a cuprous protein [127].

In contemporary times however, the ever skyrocketing technological advances have revealed much more than was initially conceived about laccases in time past, hence either corroborating what had been earlier surmised or, better still, replacing them with entirely novel tenets. Early studies on laccase focused on plant exudates and higher fungi (also discovered by Gabriel Bertrand [123]) until the discovery of the first bacterial laccase in *Azospirillum lipoferum* [100,128] with its purification and characterization [129], which was hypothesized to comprise allotments of heavy (81.5 kDa) and light chains (16.3kDA), with the latter being responsible for the holoenzyme’s catalytic activity. There has been enduring assertions since the promulgation of Enguita and colleagues’ model crystal structure of the bacterial laccase from the spore coat of the soil bacterium, *Bacillus subtilis* [130], where they inferred it to be monomeric in nature, although multimeric complexes have been so far discovered; the molecular mass of monomers may range from 35 to 135 kDa. The dimensional structure depicts it to comprise three cupric territories in which the indwelling metals are linked to different proportions of amino acid residues (approximately twelve per monomeric unit), as permitted by spatial orientation and geometry [130]. Moreover, electronic paramagnetic resonance (EPR) studies distinguished the cupric centers as types 1, 2 and 3 [131,132]. The blue colour of the protein is ascribed to the copper-cysteine bond that emits an intense electronic absorption at 600 nm, its oxidized state. Type 2 copper displays paramagnetic features but does not display recordable absorption in spectral evaluation; the type 3 copper, which is binucleated, elicits a weak electronic absorption at 330 nm, its oxidized mode, but its EPR is silent, due to anti-ferromagnetic coupling of the metal pair; type 2 and 3 copper combine as a unit to form a trinuclear cluster. However, other critical reports are beginning to pale or modify these established finds, regarding the structure of laccase. Few of such are: (i) the correction of the notion that laccases exclusively comprise copper atoms, and are blue multicopper oxidases. The discovery of the laccase variant, white laccase, from a recent studies [133,134], where the atypical colour was ascribed to the absence of the type 1 copper, and the further possession of two zinc atoms and one iron atom. Another variant, yellow laccase, possessing the same metal content as the blue laccases, but lacking the absorptions at 600 and 610 nm, pertinent to blue laccases, was discovered by Leontievsky and co-investigators [135], who reasoned that either their copper centers were distrupted in the resting enzymes’ oxidized state under aerated conditions, or the binding of low molecular mass phenolic exudates of lignin disintegration was demonstrated. On perusal of reports by Huang et al. [136], Mate et al. [137] and Daroch et al. [138], what comes to the fore is that this assertion still requires published empirical proof. In retrospect, the Bertrands could not have been absolutely wrong after all, if the enzyme is judged on the basis of ‘metallo-heterogeneity’ [123,125,126]. Thurston et al. [104] corroborate these claims. (ii) The general notion that laccases usually contain three cuprous domains per monomer could be rendered obsolete due to the emergence of two domain laccases or “small laccase” which is not uncommon with the Streptomycetes, amongst a few notable genera [139,140,141]. These unique forms of laccases have been detected to be bereft of the domain 2 [139,142], which participates in the formation of substrate- binding pocket and, subsequently, the trinuclear cluster; hence conferring a slightly different structure. The bacterial laccases examined by Feng and co-workers [143] possessed a range of 457 to 638 amino acids, although further analysis indicated that they were all intracellular. Studies on the conserved regions and conservative residues showed congruence among all the laccases studied (bacteria, plants and fungi), since they were supposedly three domain laccases. Placido and Capareda [144] who hinted that its primary structure contains approximately 500 amino acid residues arranged in three well aligned domains further surmised that there was equal splitting among the first two domains, with the third holding the largest amino acid residues. This might be due to the presence of the stabilizing disulphide bridges between the first two domains. However, reports of the number and distribution of amino acid residues in two-domain laccases remain to be seen. A distinguishing feature in bacterial laccases is the absence of a glycosylated carbohydrate fraction typical of most fungal laccases, which infamously derides the latter’s genetic manipulability and heterologous expression. Current knowledge on biochemical properties of laccase proteins emanates from model studies on purified cell extracts. Regardless of the almost indisputably high biochemical similarity of most laccases, the revelation of extensive studies have demonstrated that differences do exist not only between species, but also within species of a genera, with respect to individual strains, sampling environment, isolation and purification strategies. For example, a named bacterial strain could produce isoenzymes which possess different characteristics. However, such could only be detected through peaks and bands of column chromatography and SDS-PAGE respectively. The variations in isoforms might be ascribed to the ecological sources of the strains [145]. Furthermore, from the standpoint of Leonowicz et al. [146] they could be influenced by the culture techniques used during their production since laccases exist as inducible and constitutive forms. Some bacterial laccases, with their respective properties, have been listed (Table 1, and refrences therein). From majority of various studies carried out from the last decade to the present, it is safe to assume that bacterial laccases have a pH optimum that variee from 3 to 10; their isoelectric points may range between 4.0 and 8.4, depending on their states, respectively, and their optimal temperatures from 30 °C to 80 °C. However, it might not be exclusively so, as extracellular proteins produced by extremophiles could possess the machinery to buffer their respective activities in extreme environmental conditions. Typical examples, out of perhaps a few, are that of an Antarctic bacterium, *Pseudoalteromonas haloplanktis* [147], and a recently isolated psychrotolerant strain of *Serratia marcescens* [148]. Otherwise, most bacterial laccases have also been observed to be thermo- and alkali-tolerant; despite the putative inhibitory actions of the halides, halotolerant isoforms have been reported [149,150,151]. The universally acknowledged inhibitors thus far are sulfhydryl- containing compounds, divalent metal ions, SDS, azides, cyanides, some halides exclusive of iodide, and some highly electronegative compounds. Interestingly, Unuofin et al. [152] characterized some polyextremotelrant laccase sectretions from selected proteobacteria, whose activities were further observed to be unabated by fluoride, an otherwise renowned potent laccase inhibitor, and many other harsh reaction conditions. Conversely, the mechanism through which they manifest may depend on the structural orientation of the laccase, as well as the electrochemical nature of the substrate, on which it acts, and the respective concentrations of inhibitory molecules and the mode of inhibition portrayed by them. Three typical examples are the case of fatty acids, which could deter their binding pockets from phenolic substrates [106,153]; the halides and some other minuscular anionic inhibitors, which obstruct the reduction of molecular oxygen to water at the Type 2/3 trinuclear domain, ultimately resulting in a fall in redox potential difference between the cupreous sites, and the sulfhydryl-containing molecules, which instead reduce the already laccase-oxidized substrate [154]. Interestingly, there has been a sequence of reports on the possible reversible inhibitory activities of H_2_O_2_ ([155,156] and references therein), as determined by ABTS oxidation. Ultimately, the role of the native sources and the structural merits of laccases cannot be overshot in their interaction with these malignant substances. Seeing that most reports of laccase inhibition dwell on their compromised performances, sequel to biased attack on their cupiferous domains, a site-directed manipulation of the indwelling copper atoms should be investigated in the nearest future.

##### Substrate Specificity and Catalytic Efficiency

Since most oxidative enzymes are unmistakably driven to achieve the ultimate goal of pollution abatement at present, there remains some cynicism when considering their appositeness for real effluent treatment. This is because most municipal wastewater treatment plants, especially the centralized treatment systems, constantly receive murky and sullied fountains from heterogeneous socioeconomic hubs, which intensifies the chemical intricacies of municipal effluents. Identifiable features present therein are the cocktails of aromatic, phenolic and inorganic pollutants, which could form complexes with one another, besides harsh physicochemical conditions. This development has therefore challenged researchers and engineers alike to assess the substrate specificities of their choice “co-ferments” before subsequent environmental applications. Laccase have been, thus far, able to oxidize a memorable number of substrates (including the unlikely non-phenolic and inorganic), which could be synthetic or natural, most notably, diminutive derivatives of lignin (Table 1). Consequent of their low substrate specificity, they have achieved beyond the “centi-substrate” status [18], with an ever increasing list foreseeable. Typical examples are the reports of Reiss et al. [174] and Ihssen et al. [175], which employed about 91 substrates from different categories to assay for laccase-like multicopper oxidase activity. This outcome could be attributed to the existence of isoenzymes secreted by certain microorganisms, which might exhibit physicochemical and catalytic properties *à la carte*, yet functioning as a unit through synergism, hence their extended substrate specificity. Since laccases prefer electron-rich sources, they could be vital oxidants for aromatic rings with electron emitting groups. There however exists some disparity in classifying laccases according to their reducing substrates, since they intersect with polyphenol oxidase, bilirubin oxidase and tyrosinase, another copper-containing oxidase, based on substrate range, except the inability for laccases to oxidize tyrosine themselves [104]. Regarding reducing substrates, laccases are mostly non-specific, with their substrates range dependent on enzyme source, solvent conditions, and inevitably, the presence of free atmospheric molecular oxygen. It is then safe to assume that laccases have a broad substrate spectrum provided their redox potentials are not towering (>1 V) [106]. Apart from their strong preference for the essential oxidizing substrate, oxygen, laccases show a stronger affinity for the *para*- and *ortho*-substituted electron-laden phenol derivatives, as opposed their *meta*-substituted counterparts [176,177], where influences of steric congestion, high redox potential and net charge of the functional groups are negligible [109,178]. For example, with respect to net charge, a named substrate with electrophilic functional groups even in the *ortho* position would be less readily oxidized, as opposed to the speedily oxidized electron-laden substrates. Furthermore, Guillen and coworkers [179] were able to rationalize the higher affinity of laccases for 2,6- dimethoxyphenol (DMP) than for 2-methoxyphenol (guaiacol). This was achieved by the laccase-motivated oxidation of lignin derived hydroqinones, 2,6-dimethoxy-1,4-benzohydro- quinone and 2-methoxy-1,4-benzohydroquinone, consequently activating molecular oxygen alongside an efficient oxidation of the former over the latter. Attempts by Lante et al. [180] to degrade phenols with immobilized laccase showed that *meta*-, *ortho*- and *para*- apportioned phenols were oxidized at different rates subject to substrate concentration, in the case of *meta*-substituted ones. A plethora of studies have been conducted using 2,2′-azino-*bis*(3-ethyl- benzthiazoline-6-sulfonic acid (ABTS), an electron-dense synthetic non-phenolic compound, guaiacol, and the more laccase specific synthetic but phenolic syringaladazine, as substrates for laccase assays. However, their predilection for electron abstraction has afforded them the use of other inorganic substrates like iodide [181] and potassium ferrocyanoferrate (II) [182].

The Michaelis constant (*K_m_*) and the catalytic efficiency constant (*k_cat_*), which are a measure of the catalytic action of an enzyme, have been determined for a host of laccases and great variations have been observed among them [183,184,185,186,187,188]. The *K_m_* values of laccases may range from 2 to 500 μM, which is highly dependent on the source of enzymes, as well as the substrate applied. The lowest *K_m_* values have been measured with syringaldazine, a dimer of two molecules of 2,6-dimethoxyphenol connected by an azide bridge. The *K_m_* values of syringaldazine are generally lower, compared with those obtained with monomeric 2,6-dimethoxyphenol; the stronger affinity of syringaldazine to laccases might be due to the azide bridge or the existing dimer form. Therefore, Xu [189] pointed out that the comparison of *K_m_* values highlights the heterogeneity of substrate preferences among laccases secreted by different microorganisms. The catalytic efficiency expressed as *k_cat_* is usually characteristic of a specific protein; it represents the rate of substrate conversion to product by the enzyme per unit time. Identifiable variance has also been observed in *k_cat_* of several laccases even with the same substrates [185,186,187,190,191,192]. Laccases generally show high affinity to the non-natural substrates ABTS (non-phenolic) and syringaldazine (phenolic) with high catalytic constant, whereas their more natural counterparts, guaiacol and DMP are not rapidly oxidized, presenting comparatively higher *K_m_* values. In some cases, for example, the laccases from isolates of different ecological niches could differ markedly in their respective *k_cat_* values on the same substrate, whereas *k_cat_* values may be marginal among different substrates, per laccase monomer, further portraying the reaction rate of electron-transfer within the enzyme, sequel to substrate binding [193]. However, the kinetic constants per substrate, *sensu stricto*, should be appraised based on standard assay *modus operandi*, the solubilities of the substrates and solvents employed, enzyme purity and homogeneity, temperature, ionic strengths and pH (Table 1). Moreover, the modest understanding of the molar absorption coefficients and nature of target oxidation products and, perhaps, the interference of unreacted substrate with the enzyme-product complex or the product itself could ensure inconsistencies in *k_cat_* values. As sometimes neglected, the availability of molecular oxygen to keep the reaction seamless should always be regarded, as some assay procedures require the agitation of the mixture to keep it aerated. Ultimately, these variances should be stated when calculating and comparing catalytic constants across laboratories and reports to avoid major inconsistencies, some of which were identified by Baltierra-Trejo and colleagues [194]. Since this review addresses the aptitude of laccases in treatment of wastewater pollutants, it would be sagacious to hint that their catalytic performances should be evaluated in synthetic wastewater, and in the presence of putative inhibitors, in order to predict and subsequently tweak their responses for a fail-safe pollution cleanup. Therefore Table 1 and Table 2 have been compiled to present a conceivable depiction of the ideals that must be deliberated, when attempting the large scale treatment of real wastewater. However, real wastewater treatment process using laccase technology might be riddled with drawbacks, such as: unavailability of micropollutant for oxidative degradation in the aquatic matrix, since they are bound to the sludge, the erratic physichochemical properties of the influent, which might not be optimal for effective treatment of the heterogeneous municipal effluents. In this regard, a heuristic search for polyextremophilic proteins should be considered, and the improvement of extraction and separation techniques should be improved in the wastewater treatment plant.

##### Reaction Mechanisms and Mediator Systems of Laccases

There has been constant debate about the succinct catalysis of a variety of substrates by laccases, which has remained inconclusive, so far. Therefore, it would be worthwhile to expound what occurs *de facto* within the enzyme, when interacting with a given substrate. It has been established that laccases could assist in four substrate molecules oxidation per expended molecule of oxygen, as against the two substrates of peroxidases in a catalytic cycle. The brief comparison *infra* (Table 3) however comprises the underlying four copper atoms in three phases ordained by two pivotal domains of the laccases.

##### Substrate Binding and Oxidation Crevice: The Type 1 Copper

The characteristics of the T1 copper have been outlined *supra*. The first phase is understood to occur therein, where the catalytic cycle is initiated by the conveyance of its electrons to molecular oxygen, thereby transforming the laccase to an oxidized agitated state, and seemingly aggressively abstracts electrons from its attaching substrate to yield an oxidized product, whose radicals would initiate a cascade of other non-enzymatic activities [206]. However, it is noteworthy that the proficiency of substrate catalysis relies on differences in redox potientials between substrate and T1 copper of the laccase, which could also be affected by the pH and ionic strength of the reaction medium [207].

##### Intramolecular Electron Transport and Oxygen Reduction: Trinuclear Copper Domain (T2/T3)

After substrate oxidation in phase 1, the abstracted electrons are thereafter conveyed to the tinuclear cluster for cycle completion. Here, molecular oxygen is affixed to the T2/T3 copper center, where it accepts electrons from the copper atoms therein for its conversion to water; the laccase then reverts to its resting state. Remarkably, this electron transfer is accompanied by a proton transfer through ionizable groups within the admittance and exit conduits to the trinuclear cluster [208]. A deep insight into the mechanism will show the reader that, with respect to substrate oxidation by laccase, the phenomenon of “sowing and reaping” is seemingly highly reflected, especially from the perspective of electron exchange.

Customarily, some target compounds might not be oxidized by laccases, either due to their large size which doesn’t allow them permeation to the enzyme active site, or as a result of their remarkably high redox potential, especially when it is higher than that of the T1 copper of laccase. Another instance could be the incompatibility of functional groups required for active site recognition, which portrays a “square peg in a round hole” phenomenon. For example, oxybenzone, a personal care product, could not be directly oxidized by laccase, despite its phenolic moiety [15] Conversely, this limitation has been overcome by the discovery of ‘chemical mediators’ which serve as electron shuttles or intermediate substrates, and whose oxidized radical forms interact with high redox-potential target compounds [15]. Historically, this group of compounds was designed to avert challenges encountered in pulp bio-bleaching, by stimulating the laccase-mediated oxidation of non-phenolic compounds; thereby extending the laccase-substrate specificity ranges [209]. Mechanistically, a mediator acts as an ‘electron conveyor’ between the laccase catalytic hub and the choice substrate. Once oxidized by laccases, as always, it becomes a stable radical, the co-mediator (medox), and then rapidly diffuses away from the enzyme pocket, avoiding steric hindrances, to oxidize any substrate that could not directly enter the enzymatic pocket, by virtue of size. An interesting notion is its ability to independently oxidize substrates with high molecular weights or ionization potentials [116]. A perfect redox mediator must be a prolific laccase substrate, economically viable, with unexcitable oxidized and reduced forms that do not impede enzymatic reactions. Furthermore, redox mediators should have sufficiently high redox potentials, be regenerable and perform multiple catalytic reactions without chemical degradation [210]. A recent review by Forootanfar and Faramarzi [154] is a repository of the mechanistic purview of some redox mediators.

At present, three mediator systems have been propounded: (1) an electron transfer (ET) route for mediators, such as: ABTS, where a choice substrate is oxidized by a withdrawn electron; (2) a radical hydrogen atom transfer (HAT) route, which is prominent among the –NOH type, such as HBT and violuric acid (VIO). Here, laccase first extracts a hydrogen atom from the mediator to form a radical, this is in turn followed by a hydrogen atom conveyance from the substrate to the mediator, which is subsequently released into the medium [211,212,213], and (3) the ionic mechanism, which is characteristic of TEMPO. This compound is oxidized by laccase to its reactive oxoammonium intermediate, which oxidizes the intended substrate *via* base attack. However, it was observed that the intermediate generated deactivated the enzyme; hence the exogenous supply of a simple alcohol was suggested to scavenge the oxoammonium ion [214]. Although their mechanisms appear attractive, many of the synthetic mediators used so far are maligned by high cost, possible inhibitory effects on enzyme activity and creation of seemingly toxic metabolites, thereby motivating the search for natural forms. Laccases from unrelated organisms react differently per mediators and/or substrates [215]. Although approximately 100 unique potential mediator compounds have been enlisted for the Laccase-Mediator System (LMS), ABTS and HBT remain the most patronized [215,216]. Some natural mediators include aniline, 4-hydroxybenzoic acid and 4-hydroxybenzyl alcohol [216,217], alongside 3-hydroxyanthranilic acid (HAA), detected in the broth of *P. cinnabarinus* [218,219]. Interestingly, some of these unconventional mediators have demonstrated almost equal reliability as the commonly used ABTS and HBT [216]. Phenol red and derivatives, especially dichlorophenol red, are mediators of *Poliporus pinsitus* laccase whose oxidation quotient of the nonphenolic substrate 4-methoxybenzyl alcohol was reported to be at least 10 times higher than with HAA [217]. Morozova et al. [210] hinted that most compounds are not worthy “laccase mediators” as ascribed, due to their electrochemically unsteady oxidized intermediates, thereby eliciting a meager amount of redox cycles per catalytic oxidation of non-phenolic compounds. Consequent of this, the term ‘enhancer’ has sufficed for a more accurate definition. There has been no aggressive employment of LMS at large scale due to the earlier stated reasons. However, the adoption of naturally-occurring laccase mediators would present the “light at the end of the tunnel” for *in situ* environmental applications. In congruence with this hope, Camarero et al. [220] recounted that numerous lignin-derived phenols (such as syringaldehyde and acetosyringone) heralded ecofriendly alternatives to synthetic mediators for the disintegration of dye assortments and recalcitrant xenobiotic substances by laccase from perspective of efficiency and oxidation velocity. Interestingly, the discovery of the ability of a special form of laccase, the ‘yellow/white’ laccase, to oxidize non-phenolic and high redox potential substrates in the absence of mediator substances innately required by blue laccases, has been reported by Leontievsky et al. [135], who observed that its production was a function of media composition. This implies that the search for novel laccase with this special catalytic feature might outpace the need for natural mediators in the nearest future. Furthermore a heuristic search should be conducted for substrates, which can both serve as feedstock for novel laccase production, as well as excellent mediators, in order to achieve a remarkable economy of production.

##### Laccase in Wastewater Treatment: A Prospective Stance

Irrespective of their high volumetric quotient, only a fraction of effluents generated by the industries receive satisfactory treatment before final discharge into natural water bodies. Wastewater ordinarily contains mixtures of effluents, which vary in physico-chemical attributes, oftentimes degradation resistant, leading to constant loading and coercion of deleterious effects on the aquatic life [221,222,223]. This has prompted the need for stringent measures, as regards the daily discharge limit initiative, which had been set as total maximum daily load (TDML) in the United States [224], the integrated pollution prvention and control (IPPC) amongst the European Union [225] and the minimum acceptable standards (MINAS) for industrial and municipal discharges in India [226]. However, as this requirements look technically unattainable, the prospect of appropriate treatment technologies becomes imperative.

**Figure 4:**
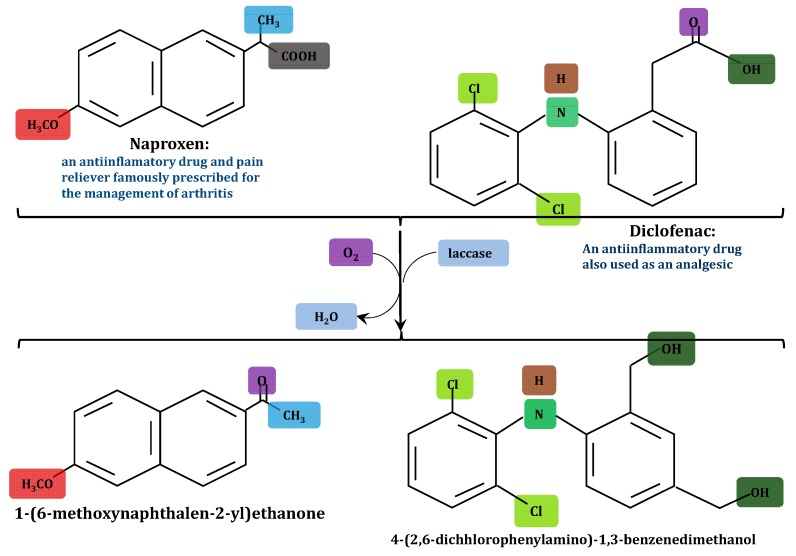
Laccase oxidative transformation of some hospital wastewater pollutants.

**Figure 5:**
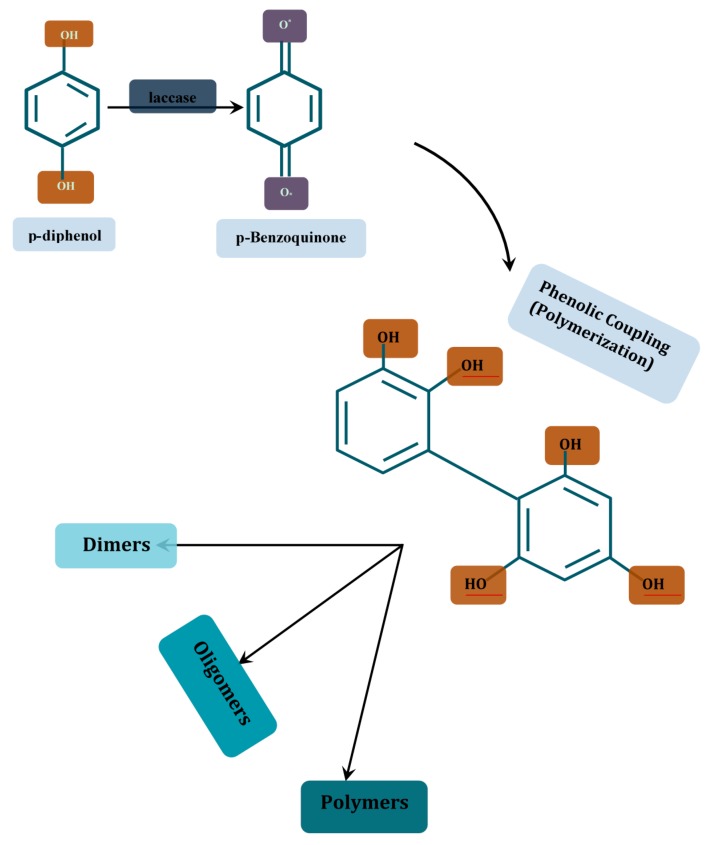
The polymerization of phenolic radicals generated from xenobiotics degradation.

The catalytic properties of laccases earlier discussed make them credibly suitable for a broad range of environmental clean-up strategies. There is ample data on the biotechnological applications of laccase, but regarding this review, preference is directed towards their degradation potentials. First, the propensity of laccase-catalyzed wastewater treatment has been extrapolated from preparatory scale outcomes of model studies which include, though not limited to, the following: the two-step oxidation of alkenes, phenols and aromatic amines to ketone or aldehyde [227,228]; laccase treatment of UV filter and various halogenated pesticides [107]; dye decolorization [229,230,231]; laccase-based delignification of woody and non-wood plant feedstocks [232] and the degradation of pharmaceuticals (Figure 4) [109,233,234,235]. The mechanistic perspective of the the above applications however involves the enzymatic oxidation of the pollutants, especially those with electron-donating groups, to free radicals or quinones, which subsequently undergo polymerization and partial precipitation (Figure 5) [106,227]. Furthermore, with respect to the above stated model studies, it is foreseeable that laccase-catalyzed xenobiotic pollutant elimination from wastewater can be executed in the following methods: (1) using crude free laccases; (2) using purified free laccases; (3) using immobilized crude or purified laccases; (4) using immobilized or free laccase producing cells in bioremediation reactors; and (5) the syngergy of redox mediators with any of the four methods.

**Figure 6:**
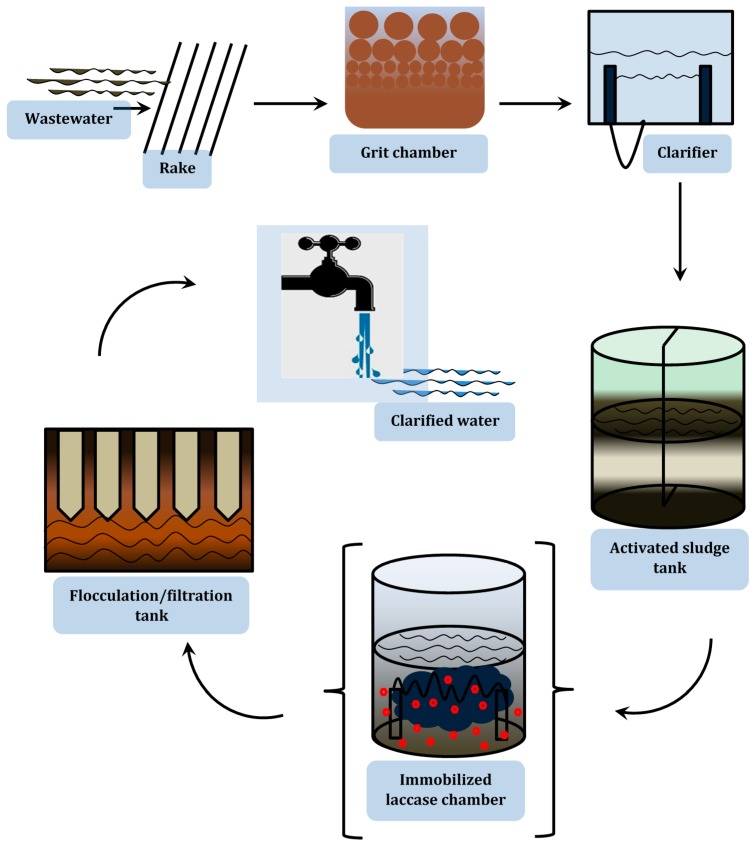
Schematic of a typical conventional wastewater treatment process showing the prospective strategic position of laccase treatment. The laccase immobilization chamber comprises a pretreated lignocellulosic support, which possesses antimicrobial properties. The location of the chamber helps to reduce or totally eliminate aromatic pollutants that could interfere with the efficiency of the flocculation step.

Since the claims of Majeau et al. [178] and Zimmermann et al. [236] that laccases could be considered as relatively stable enzymes, with half-lives of several days in treated municipal wastewater at 20°C, corroborative studies that purport the feasibility of laccases in treatment of real wastewater include the laboratory- and bioreactor-scale treatments of some industrial effluents. In a 2015 study carried out on wastewater from a textile factory near Cairo, Egypt, Gomaa and Momtaz [237] achieved 71% decolorization when laccase was applied individually, and subsequently established a laccase-copper synergistic effect for further improvement in textile wastewater treatment. Ba et al. [238], during an eight-hour study, achieved near 100% transformation of acetaminophen in municipal and hospital wastewater samples, respectively, through treatment with cross-linked laccase aggregates (CLEA); similarly, Arca-Ramos and colleagues [239] still recorded about 95% transformation of bisphenol A (BPA) despite recording a laccase activity below target concentration in real secondary effluent, using an enzymatic membrane reactor. A recent laccase-catalyzed treatment of real wastewater cocktail showed not only a high biodegradation rate of most of the pollutants, but also an appreciable reduction in their ecotoxicity [240]. Laccases have been used to remove chlorophenols and chlorolignins from kraft bleach effluents [241]. Other salient contributory studies include works done by Dellamatrice et al. [242] Steevensz et al. [243], Du et al. [244], Lloret et al. [30] and Nair et al. [245]. Noticeably, Li et al. [246] were able to effectively remove oil from a petroleum-spiked synthetic wastewater by an immobilized laccase catalyzed oxidation, whereas an increase in concentration of pharmaceutically active compounds correspondingly increased their rate of laccase-catalyzed transformation in wastewater. This was also concurrent with the formation of cross-coupling products, which were examined to be interestingly novel mediators for laccase catalysis [247]. Regardless of the relative successes recorded in previous studies, some intricacies are traditionally encountered, such as: the discrepancies in optimal substrate pH for treatment, where a compromise between laccase stability and its activity is considered for optimal treatment pH [111]; differences in reactivity, which could be related to differences in chemical composition and orientation of the substrates [248,249]; disturbance of proper enzyme configuration by unfavourable chemico-physical conditions [240]; interruption of enzyme catalysis on target pollutants by suspended particles, heavy metals, colloids, salts solvents and some autochthonous microflora atypical of real wastewater [145,240]; and the overwhelming need of large quotients of enzymatic activity for complete removal of pollutants [23]. These have made the prospect of their full adoption by various wastewater treatment facilities seem somewhat unappealling. In this regard, we are conscious that the structural configuration of laccases, in tandem with their strategic positioning in any given wastewater treatment setting, would be crucial for their overall performance. So far, possible enzyme structural configurations and their respective permutations, with other auxiliary supports in several wastewater treatment schemes have been critically appraised by Jun et al. [250] Arca-Ramos et al. [251], Singh et al. [252], and references therein. Consequently, deductions made from their critical evaluations depict the legitimacy of the adsorption of laccases on membranes to carry out the integrated aim of micropollutant degradation, and inhibition or extermination of harmful microorganisms and viruses. However, with regard to a conventional wastewater treatment scheme, the positioning of the laccase reaction tank, which might contain laccase adsorbed on economically feasible supports like biochar, coconut coir and nanoclay, could be crucial to the overall residual toxicity and clarity of the wastewater, provided it is situated before the flocculation step as portrayed in Figure 6. This recommendation is directed at the financially incapacitated communities who are visibly struggling to meet up with demands of the state-of-the-art-technologies. Our ratiocination was substantiated by earlier investigations by Rebhun and Galil [253], as well as Rozich et al. [254], who observed that shock loads of phenolic and aromatics substrates may potentially create a bottleneck to the bioflocculation process. Notwithstanding, the achievements of the IGEM Team Bielefeld-Germany project in 2012 [255] and other notable studies displayed in Table 2 present an epitome of real life wastewater treatment majority of researchers and biotechnological industries should aspire to in the nearest future. Le et al. [256] were able to achieve simultaneous degradation of triclosan and recalcitrant dyes in real wastewater using laccase encapsulated in core-shell magnetic copper alginate beads. Interestingly, a multipurpose residue was formed when nanoclay was employed in the bleaching of indigo denims and the successive decolorization of the highly coloured effluent [257]. The residue was suggested to be ornamental, and could be employed as coatings and plasters for walls, which would be an epitome of total sustainability of the environomics of the treatment of textile industry effluents.

##### Life Cycle Assessment of Laccase Production and Application: Beyond Technoeconomics

Ever since the production of the foremost industrial laccase “DeniLite^®^”, the earliest bleaching enzyme whose action was facilitated by a mediator molecule, by Novozymes (in 1996), other biotechnological and chemical industries have pursued this precedent, consequently manufacturing enzymes which are expensive and not fiscal for large scale applications. The majority of laccases commercially produced so far are of fungal origin, though some have been reported to have less activity, in free or immobilized states, compared with crude enzyme extracts from other fungi [258,259]; a phenomenon left indecipherable. Interestingly, the commercialization of a bacterial laccase Metzyme (Cat-No: 10-101-UF), which has tremendous ligninolytic potentials and robust catalytic properties, by MetGen, a biotechnology company based in Kaarina (Finland), has inspired a lot of studies toward the scale-up and the commercialization of other bacterial laccases in no distant future. However, as earlier mentioned, the critical analysis of the overhead involved in the time line of laccase production on laboratory scale makes its prospects of commercialization unattractive. A typical example could be inferred from Table 4, which is the cost analysis of consumables required for successful screening and production of bacterial laccases.

Moreover, thinking beyond technoeconomics, the environomics of the laccase production would not be entirely beneficial, with regard to the model portrayed in Table 4, because high consumption of chemicals is involved. These chemicals might, in turn, contribute to environmental pollution (most especially eutrophication of aquatic matrices), once the spent fermentation medium for a particular batch is disposed. Therefore, in order to monitor and improve the environomics of a production process, Roger Sheldon introduced a green chemistry metric, the E(nvironmental) factor (*E* factor), which appraised the total amount of waste (kg) generated per specific product (kg), and the overhead of all raw materials enacted in the process [260]. In the light of these, it cannot be disputed that the synthesis of metabolites by the aforementioned chemicals would not be a desirable end, on the largescale. Correspondingly, the application of these enzymes using expensive mediators like ABTS would not translate into covetable returns, since the public is becoming increasingly aware of environmental pollution, and the implications of use of environmentally austere chemicals. To this end, cost effective and environmentally benign approaches are craved for, and must be encouraged. Interestingly, studies have recently been conducted, which point to the beneficial production of laccase from environmental wastes. Unuofin et al. [261] had proposed the adoption of wastewater as a repository for robust laccase producing bacteria, after some of its bacterial denizens produced laccase that exhibited interesting catalytic potentials. This was followed by the demonstration of laccase production in copious amounts from some agroindustrial residues, using statistical models [262,263]. It was discovered in the studies that some aromatic compounds like 4-nitrophenol and acetaminophen induced laccase production, which implies the dilemma of cost-intensive production and environmental pollution could be collectively solved, especially if these agricultural wastes are humidified with wastewater, which expectedly contains varying amounts of these aromatic pollutants. Consequently, in compliance with the ‘zero waste policy’, some of the fermented agroindustrial wastes could be applied as biofertilizer, independently, or as a ‘mulch mix’, since their integral basic nutrients, like organic nitrogen and potassium, might have been unlocked during their degradation. Similarly, the application of laccase in real wastewater treatment would be environomically feasible, if they adopt greener materials such as pretreated agroindustrial wastes like coconut coir and other lignocellulosic carriers [264], as well as polymers synthesized by bacteria [265]. Although some transformation products of the laccase catalysis might be potentially undesirable, just as is fondly observed with chemical oxidation, a state-of-the-art characterization of these residues would be beneficial, as it would guide technologists and engineers to the most suitable processes, in which they could be adopted as starter chemicals. Moreover, a site-directed evolution approach to enzyme technology would be a major player in the avoidance of aggressive enantioselectivity during laccase-catalyzed reactions.

## 4. Conclusions

In a perfect world, laccases will undoubtedly be the usual suspects, when considering biocatalysts for environmental applications, since they require only the almost readily available co-substrate, “atmospheric molecular oxygen”, as a vehicle for either the cross-linking of monomers, degradation of often recalcitrant aromatic polymers and other otherwise constitutive oxidations, which all partake in the maintenance of seamlessness of the environmental and the socioeconomic cycle. It is quite interesting to note that some derivatives of the polymer breakdown could constitute bulding blocks of other industrially fine chemicals since the rate the rate of polymer catalysis might be proportional to monomer cross-linkage (in a batch system); this aspect will be expounded in a forthcoming review. It is therefore recommended that further batch studies and criticial analyses of analytes should be conducted, and the chromatogram of all derivatives characteristic of laccase-substrate interactions should be elucidated to provide useful information to enable the apposite and uttermmost benefits from these reactions on a wide scale.

Although laccases are at present still produced in limited quantities, their potential is inexhaustible, as many still remain to be uncovered whilst relative successes are being recorded in some aspects of wastewater treatment. Surreally, despite all these discoveries, the employment of biological processes using laccase based biotechnology might not be getting corresponding attention because of their relatively high production costs, low yield, low stability under some pertubingly harsh indsutrial or environmental conditions. The limitations listed so far can be adequately addressed by the following: (i) favourable exploitation of microbes for enhanced laccase production through the identification and characterisation of genomic sequences coding for laccase production and subsequently the application of strain improvement techniques; (ii) Optimization of production conditions, which will include the formulation of several cheap substrates like certain agroindustrial and environmental wastes, that will stimulate the production of yellow laccases; (iii) the expansion of the spectrum of natural, renewable, cheap and ecofriendly mediators which would promote real life applications of laccases, and lastly (iv) The investigation of optimal process conditions and the most suitable economical viable and environmental-friendly supports for immobilization of the laccases, which will enhance their durability and reusability, hence presenting an environomically feasible approach to wastewater treatment. These fall in line with the primary objective of any treatment system which is “to minimize cost as well as environmental pollution”.

## Figures and Tables

**Table 1:** Physicochemical and catalytic properties, and substrate specificities of some bacterial laccases.

Specimen Studied/Origin	Molecular Weight (kDa)	pH Optima/Substrate	Temperature Optima (°C)	*K*cat/*K*_m_ (mmol/(L/s))	Activity Enhancers/Inhibitors	References
*Aeromonas hydrophila* WL-11/Textile effluent	59	2.6/ABTS, DMP/8.0	40,50	87.21 (ABTS) 112.56 (DMP)	Cu^2+^/Ca^2+^	Wu et al. [157]
Cot A lac from *Bacillus* sp. HRO3 from Iranian microflora	66.2	4.0/ABTS, 7.0/SGZ, 7.4/DMP	70 (ABTS) 70,80 (SGZ)	0.24 (ABTS) 4 (SGZ) 0.06 (DMP)	NaCl (inhibitor)	Mohammadian et al. [158]
*Streptomyces ipomea*	77	5.0/ABTS 7.5-8.0/SGZ 8.0/DMP	60	2.50 × 10^4^ (ABTS)	KCN (inhibitor)	Molina-Guijarro et al. [159]
*Bacillus pumilus*	58	4.0/ABTS 6.5/SGZ 7.0/DMP	55-75 (ABTS) 70 (DMP)	3.64 (ABTS) 0.02 (DMP)	ND	Reiss et al. [119]
*Corynebacterium efficiens* from maize rhizosphere	ND	9.0/ABTS	70 (ABTS)	1015.4 (ABTS)	K^+^, Na^+^	Odeniyi et al. [160]
*Bacillus licheniformis* LSO4 spore laccase from chinese soil	ND	4.2/ABTS 6.6/SGZ 7.4/DMP	60	ND	Inhibitors: Dithiothreitol, sodium azide, l-cysteine	Lu et al. [161]
Lac21 from South China Sea marine microbe	50	7.0/Catechol 6.5/K_4_Fe(CN)_6_ 7.5/SGZ 8.0/DMP	45	1.26 × 10^6^ (SGZ) 8.25 × 10^4^ (DMP) 2.66 × 10^5^ (catechol) 8.42 × 10^5^ (K_4_Fe(CN)_6_	Enhancers: NaCl, K_2_SO_4_ Inhibitor: EDTA, Co^2+^	Fang et al. [149]
*Bacillus amyloliquifaciens*	65	3.8/ABTS 6.6/SGZ	60 (SGZ)	0.41 (SGZ) 0.16 (ABTS)	Enhancers: NaCl Inhibitors: NaN_3_	Chen et al. [162]
*Shigella dysenteriae*	55	2.5/ABTS	25	211	Enhancers: Cu^2+^ Inhibitors: EDTA, NaN_3_, Thioglycolic acid.	Shao et al. [163]
*Streptomyces* sp. SB086 from Brazilian reforestation soil	≥ 100	4, 5/ABTS	50	ND	Enhancers: Cu^2+^, K^+^, Mg^2+^, Zn^2+^ Inhibitors: Fe^2+^	Fernandes et al. [164]
*Lactobacillus plantarum* J16 CECT 8944	62.5	3.5/ABTS 7.0/DMP	60 (DMP)	*K*_m_: 0.21 mM (ABTS) 1.67 mM (DMP)	Inhibitors: bipyridyl, EDC, phenanthroline	Callejón et al. [165]
*Meiothermus ruber*	50	5.0/ABTS 7.5/SGZ 8.0/DMP	70 (ABTS)	0.19 (ABTS) 0.42 (SGZ) 0.63 (DMP)	Enhancers: Cu^2+^, Mn^2+^, Ba^2+^ Inhibitors: Ni^2+^, Mg^2+^, Fe^2+^, Zn^2+^	Kalyani et al. [166]
*Corynebacterium glutamicum*	59	7.5/ABTS, SGZ, DMP	60	44.8 (ABTS) 125.8 (SGZ) 33.0 (DMP)	Enhacer: Acetone Inhibitor: DMSO.	Ricklefs et al. [167]
*Streptomyces sviceus*	32.5	4.0/ABTS 8.0/SGZ 9.0/GUA, DMP	ND	1230.56 (ABTS) 0.22 (SGZ) 21.68 (DMP)	Enhancer: Hexane Inhibitors: Water miscible co-solvents	Gunne et al. [168]
*Haloferax volcanii* US02	75-80	6.0/ABTS 8.4/SGZ	45-50	0.015 (ABTS) 0.62 (SGZ)	Enhancers: Inhibitors: NaN_3_, thiourea	Uthandi et al. [169]
*Thermoalkalivibrio* sp.	60	5.0/ABTS >9.5/DMP	50	0.475 (ABTS) 0.00375 (DMP)	Enhancers: K_2_SO_4_, Na_2_SO_4_	Ausec et al. [170]
*Paenibacillus glucanolyticus* SLM1	90	7.0/ABTS	40	36.1 (ABTS) 0.75 (CAT) 8.85 (DMP)	NA	Matthews et al. [171]
*Streptomyces coelicolor* A3(2) SLAC and variants	90	4.0–4.5/ABTS 7.0–10/DMP	90–95	0.723–1.874 (ABTS) 0.900–2.226 (DMP)	Inhibitors: NaN_3_, SDS, EDTA.	Prins et al. [141]
*Bacillus tequilensis* SN4	32	5.5/ABTS 8.0/DMP 6.5/SGZ 8.0/GUA	85	47.82 (ABTS) 87.05 (DMP) 19.34 (GUA)	Inhibitors: β-mercaptoethanol, Dithiothreitol, Cysteine.	Sondhi et al. [172]
*Thermus thermophilus*	53	4.5/ABTS 6.0/SGZ 7.5/GUA 8.0/DMP	90 (GUA)	0.011 (ABTS) 0.012 (GUA) 0.037 (SGZ) 0.001 (DMP)	Enhancer: K^+^, Li^+^ Inhibitors: EDTA	Liu et al. [150]
*Thermus thermophilus*	27	4.5/ABTS 6.0/SGZ	75 (ABTS)	3.02 (ABTS) 5.14 (GUA) 8.75 (SGZ) 26.63 (DMP)	Inhibitors: NaN_3_, EDTA, topolone	[173]

**Table 2:** The treatment of different wastewater pollutants in aqueous systems by some laccase.

Pollutant	Rate of Removal (%)	Enzyme Orientation	Source	References
Acetaminophen	95	Cross-linked laccase aggregates and polysulfone hollow fiber microfilter membrane	*Trametes versicolor*	Ba et al. [195]
Mefenamic acid				
Carbamazepine				
Estrone	83.6	Laccase adsorbed on enzymatic membrane reactor	*Myceliophthora thermophilia* (commercial)	Lloret et al. [196]
17β-estradiol (E2)	94			
17α-ethinylestradiol (EE2)	93.6			
Bisphenol A	100	Crude laccase	*Pleurotus pulmonarius*	de Freitas [197]
Bisphenol A	80	Immobilized laccase	*Cerrena unicolor*	Songulashvili et al. [198]
Nonylphenol	40			
Triclosan	60			
Bisphenol A	89	Free laccase amalgam	*Pycnoporus sanguineus* CS43	Garcia-Morales et al. [199]
4-nonylphenol	93			
17α-Ethinylestradiol	100			
Triclosan	90			
Bisphenol A	≥100	Immobilized laccase	*Trametes versicolor*	Zdarta et al. [200]
Bisphenol F	≥100			
Bisphenol S	>40			
Bisphenol A	90	Immobilized laccase	*Pleurotus ostreatus*	Ji et al. [201]
Carbamazepine	40			
Tetracyclines	100	Immobilized laccase Immobilized laccase + ABTS	*Myceliophthora thermophile*, *Pleurotus eryngii*	Garcia-Delgado et al. [202]
Sulfathiazole	100			
Sulfadiazine	54			
Bisphenol A	100	Free laccase	*Bacillus* sp. GZB	Das et al. [203]
Chlorpyrifos	98.7	Surface immobilized laccase	*Pseudomonas putida* MB285	Liu et al. [204]
Bisphenol A	Cca.88-100	Crude laccase	*Streptomyces cyaneus*	Margot et al. [205]
Diclofenac	Cca.60-cca.85			
Mefenamic acid	Cca.50-cca.95			
Triphenylmethane dyes	Cca.68-CCa.71	Crude laccase	*Corynebacterium efficiens*, *Enterobacter ludwigii*	Odeniyi et al. [160]

**Table 3:** Brief comparison of laccase/peroxidase-substrate oxidation per catalytic cycle.

Laccase	Peroxidase
(Aromatic ↔ Aromatic * + e^−^)	(Aromatic ↔ Aromatic * + e^−^)
(4e^−^ + 4H^+^ + O_2_ ↔ 2H_2_O)	(2e^−^ + 2H^+^ + H_2_O_2_ ↔ 2H_2_O)
4 Aromatic + 4H^+^ + O_2_ ↔ 4Aromatic * + 2H_2_O	→ 2Aromatic + 2H^+^ + H_2_O_2_ ↔ 2Aromatic * + 2H_2_O

* = oxidised aromatic substance.

**Table 4:** Cost of selected chemicals for production of inoculum and microbial fermention.

S/N	Name	Quantity	Amount (ZAR)	Company and CAS No
1	Bacteriological Agar	1 kg	10,740.00	Sigma-Aldrich (A5306-1KG)
2	Citric Acid	500 g	980.00	Sigma-Aldrich (251275-500G)
3	K_2_HPO_4·_3H_2_O	500 g	1082.00	Sigma-Aldrich (P5504-500G)
4	LB Broth	1 kg	1914.00	Sigma-Aldrich (L3022-1KG)
5	Luria Agar Base	1 kg	8140.00	Sigma-Aldrich (L2025-1KG)
6	Glycerol	100 mL	668.00	Sigma-Aldrich (G5516-100ML)
7	NaCl	500 g	551.51	Sigma-Aldrich (746398-500G)
8	CuSO_4·_5H_2_O	250 g	929.35	Sigma-Aldrich (209198-250G)
9	CaCl_2·_2H_2_O	500 g	539.78	Sigma-Aldrich (223506-500G)
10	FeCl_3·_3H2O	250 g	1039.67	Sigma-Aldrich (220299-250G)
11	FeSO_4·_7H_2_O	250 g	478.76	Sigma-Aldrich (215422-250G)
12	(NH_4_)_2_SO_4_	500 g	1032.00	Sigma-Aldrich (A4418-500G)
13	MgCl_2·_6H_2_O	100 g	642.00	Sigma-Aldrich (M2670-100G)
14	NH_4_Cl	500 g	552.00	Sigma-Aldrich (A9434-500G)
15	Syringaldazine	1 g × 6	9456.00	Sigma-Aldrich (S7896-1G)
16	ABTS	10 mg (50 tablets)	3280.00	Sigma-Aldrich (A9941-50TAB)
17	Guaiacol	250 g	916.00	Sigma-Aldrich (G5502-250G)
18	Vanillin	100 g	360.00	Sigma-Aldrich (V1104-100G)
19	2,5-Xylidine	100 mL	326.00	Sigma-Aldrich (102253-100ML)
20	CoCl_2_.6H_2_O	100 g	1729.65	Sigma-Aldrich (255599-100G)
21	Yeast Extract	250 g × 2	1916.00	Sigma-Aldrich (Y1625-250G)
22	Alkali lignin	500 g	2920.00	Sigma-Aldrich (471003-500G)
23	MnSO_4·_H_2_O	100 g	724.00	Sigma-Aldrich (M8179-100G)
		Total	48,691.37	
